# A Polysocial Approach in Exploring Racial and Ethnic Differences in Dementia and Cognitive Decline Among U.S. Older Adults: Health and Retirement Study

**DOI:** 10.1093/geroni/igae078

**Published:** 2024-08-28

**Authors:** Yongjing Ping, Michelle C Odden, Xi Chen, Matthew Prina, Hanzhang Xu, Hao Xiang, Chenkai Wu

**Affiliations:** Program in Health Services and Systems Research, Duke-NUS Medical School, Singapore, Singapore; Global Health Research Center, Duke Kunshan University, Kunshan, Jiangsu, China; Department of Epidemiology and Population Health, Stanford University, Stanford, California, USA; Department of Health Policy and Management, Yale School of Public Health, New Haven, Connecticut, USA; Department of Economics, Yale University, New Haven, Connecticut, USA; Population Health Sciences Institute, Faculty of Medical Sciences, Newcastle University, Newcastle upon Tyne, UK; Program in Health Services and Systems Research, Duke-NUS Medical School, Singapore, Singapore; Department of Family Medicine and Community Health, School of Nursing, Duke University, Durham, North Carolina, USA; Department of Global Health, School of Public Health, Wuhan University, Wuhan, Hubei, China; Global Health Institute of Wuhan University, Wuhan University, Wuhan, Hubei, China; Global Health Research Center, Duke Kunshan University, Kunshan, Jiangsu, China

**Keywords:** Health disparities, Longitudinal data analysis, Social determinants of health

## Abstract

**Background and Objectives:**

The racial or ethnic disparity in the burden of dementia exists among older adults in the United States, whereas gaps remain in understanding the synergic effect of multiple social determinants of health on diminishing this disparity. We aim to build a polysocial score for dementia and investigate the racial or ethnic difference in dementia risk among older persons with different polysocial score categories.

**Research Design and Methods:**

In this prospective cohort study, we utilized longitudinal data from the Health and Retirement Study in the United States recruiting 6 945 participants aged ≥65 years who had data on 24 social determinants of health in 2006/2008. The dementia status of participants was measured by a modified version of the Telephone Interview of Cognitive Status. The stepwise Cox regression was applied to select social determinants of health associated with incident dementia to construct a polysocial score. The multivariable Poisson model and linear mixed model were utilized to investigate the associations between polysocial score and incident dementia and cognitive decline, respectively.

**Results:**

Eight social determinants of health were used to build the polysocial score. Non-Hispanic Black older participants had a higher incidence rate (incidence rate difference [IRD] = 22.7; 95% confident interval [95% CI] = 12.7–32.8) than non-Hispanic White older adults in the low polysocial score, while this difference was substantially attenuated in the high polysocial score category (IRD = 0.5; 95% CI = −6.4 to −7.5). The cognitive decline of non-Hispanic older Black adults with high polysocial score was 84.6% slower (averaged cognitive decline: non-Hispanic White: −2.4 [95% CI = −2.5 to −2.3] vs non-Hispanic Black: −1.3 [95% CI = −1.9 to −0.8]) than that of non-Hispanic older White persons.

**Discussion and Implications:**

These findings may help comprehensively understand and address racial and ethnic disparities in dementia risk and may be integrated into existing dementia prevention programs to provide targeted interventions for community-dwelling older adults with differentiated social disadvantages.

**Translational Significance**: We developed a polysocial score that incorporates individual- and community-level social factors to address racial and ethnic disparities in dementia incidence among multiethnic older adults in the United States. Increasing the polysocial score significantly reduced the gap in dementia risk between non-Hispanic White and Black older adults. Social factors were weighted by their associations with dementia risk, helping identify key social factors for reducing dementia risk. This approach may guide effective preventions tailored to improve cognitive function and reduce dementia risk through enhancing the social environment, benefiting older adults most in need.

## Background and Objectives

The number of older adults, including those living with dementia, is increasing rapidly in the United States. In 2021, an estimated 6.2 million older Americans are affected by dementia (1). This number is projected to grow to 13.8 million by 2060 (1). Although the age-standardized incidence of dementia has gradually declined in the United States over the past several decades, racial and ethnic disparities in dementia continue to be a public health concern (2,3). Epidemiological studies have consistently shown a higher burden of dementia among racial and ethnic minorities, especially non-Hispanic Black older adults (4–6). Reducing racial and ethnic disparities in dementia has been set as a national priority by the National Alzheimer’s Plan (7).

Prior research demonstrated that multiple social determinants of health, such as education, health literacy, household income, and financial adequacy, can contribute to the racial and ethnic disparities in dementia risk independently (8–11). However, prior studies have not adequately addressed that social determinants of health are multidimensional and may cumulatively affect individual cognitive function (8,12). Healthy People 2030 classifies the social determinants of health into 5 domains: economic stability, education, healthcare, neighborhood and built environment, and social and community context (13). Inspired by the polygenic risk score, which quantifies the aggregated effects of genetic variants, the concept of polysocial score was recently proposed to capture the aggregated effect of multiple social determinants of health on distinct health outcomes among older adults (14–16). This novel approach may help comprehensively understand and address racial and ethnic disparities in dementia among older U.S. adults.

The aim of our study was twofold. First, we created a polysocial score for incident dementia in a nationally representative sample of community-dwelling older adults in the United States. Second, we examined the moderative effect of polysocial score between race or ethnicity and the incidence of dementia as well as cognitive decline. We hypothesized that (1) a higher polysocial score is associated with a lower incidence rate of dementia and a slower decline of cognitive function and (2) the racial and ethnic differences in dementia incidence rate and cognitive decline will be attenuated by increasing polysocial score among older adults aged 65 and above in the United States.

## Research Design and Methods

### Setting and Participants

The Health and Retirement Study (HRS) is an ongoing longitudinal cohort study of a nationally representative sample of noninstitutionalized residents aged 50 years or above in the contiguous United States. The HRS aims to describe changes in life patterns through the retirement transition among U.S. adults by collecting information about their health, family network, social relations, finance, and employment status (17). Ethical approval was obtained from the University of Michigan Institutional Review Board. Written informed consent was collected from all participants. Details on study design, recruitment protocol, and data content could be found elsewhere (17).

The HRS core survey was conducted biennially since 1992 and had a response rate of >80% in each follow-up. Since 2006, approximately half of the HRS participants were randomly selected to complete a left-behind psychosocial questionnaire that participants returned by mail. The psychosocial questionnaire collected information about living conditions, subjective well-being, lifestyle, the experience of stress, and social relationships. The other approximately half of the participants were given the same questionnaire in the next interview wave in 2008.

### Sample

We included 12 836 participants who completed the additional psychosocial questionnaire in either the 2006 or 2008 wave. We then excluded 4 469 participants under 65 years of age, 257 participants with dementia at baseline, 778 participants with missing information of employment status, 120 participants without data on household safety, 12 participants without censoring date, 74 participants with missing data on insurance coverage, 72 participants without cognitive tests, and 109 participants whose race or ethnicity were unclear. A total of 6 945 participants were included in the final analytical sample. A flow diagram was presented to illustrate the detailed inclusion process (see Supplementary Figure 1).

### Social Determinants of Health

We included 24 social determinants of health encompassing 5 categories: economic stability, education, healthcare, neighborhood environment, and social and community context at baseline to capture a comprehensive individual-level social risk following the same selection strategy we previously developed (18). Detailed information on these social determinants of health is listed in Supplementary Table 1.

### Cognitive Function and Dementia

Cognitive function was assessed by a modified version of the Telephone Interview for Cognitive Status (TICS), a validated cognitive assessment following the Mini-Mental State Examination for complex population-based longitudinal studies, in the HRS (19). The TICS consists of the immediate word recall test and the delay word recall test for episodic memory evaluation, serial 7’s test and backward counting test starting from 20 and 86 for working memory testing, and naming tests to examine participants’ memory of date, listed objects, and current president or vice president (19). We adapted the composite score consisting of the immediate (0–10) and delayed (0–10) word recall test, serial 7’s test (0–5), and backward counting test (0–2), ranging from 0 to 27 (20).

The primary outcome of this study is the incidence of dementia, defined as participants with a score of 0–6 in the earliest one of follow-up waves; those with a score of 7 or above throughout the follow-up periods were considered not to have incident dementia. The validity and reliability of this cut-point for dementia diagnosis among U.S. community-dwelling older adults were shown to be adequate (21,22). The secondary outcome was the temporal change in cognitive function. We utilized the TICS as a continuous variable to measure the longitudinal change in cognitive function of participants from 2006 to 2016.

### Covariates

The demographics, lifestyles, and health conditions of participants were assessed in either 2006 or 2008 depending on the year of completing a psychosocial questionnaire in the HRS. Demographics included age in years, sex, and race or ethnicity (non-Hispanic White, non-Hispanic Black, and Hispanic). Lifestyles included body mass index (BMI, kg/m^2^), calculated as body weight in kilograms divided by standing height in meters squared and classified into underweight or normal (BMI: ≤24.9), overweight (BMI: 25.0–30.0), and obese (BMI: ≥30.0), smoking status (never, former, and current), and alcohol consumption (0, 1–2, 3–4, and 5+ drinks per week). Health conditions were self-reported by participants in 2006 or 2008, including disability in activities of daily living (ADLs), defined as having difficulty in performing any one of the 5 ADLs (bathing, dressing, eating, getting in/out of bed, and walking across a room), hypertension, diabetes, cancer, lung disease, heart disease, stroke, psychiatric disease, and arthritis. Self-rated general health was classified as excellent/very good, good, and fair/poor.

### Statistical Analyses

We used the forward stepwise Cox regression, with a *p* value of .10 as the threshold (23), to screen for important social determinants for incident dementia. Retained social determinants of health were used to construct the polysocial score. To assign a score for each retained variable, we used their coefficients from the stepwise Cox regression multiplied by 10 to calculate an integer number for all retained social determinants of health, reflecting the strength of their associations with incident dementia (24,25). These scores were then summed to create a polysocial score to evaluate individual-level overall social risk of incident dementia, with a higher polysocial score indicating a lower risk of incident dementia. We also provided a categorical polysocial score, classified into low (0–20), intermediate (21–27), and high (28+) based on its tertiles.

We described the bivariate associations between the demographic, lifestyle, and health characteristics of participants and categorical polysocial score using analysis of variance (ANOVA) for continuous variables and the chi-square test for categorical variables. We calculated the overall and race/ethnicity-specific incidence rates of dementia (cases per 1 000 person-years [PYs]) by polysocial score categories. The Poisson regression was applied to estimate the association between polysocial score and incident dementia; we then tested the additive interaction between polysocial score and race or ethnicity. We used the linear mixed model to delineate temporal change in cognitive function by polysocial score categories. We also estimated the race- or ethnicity-specific longitudinal trend of cognitive decline in the linear mixed model. The following covariates were considered as potential confounders in the adjusted Poisson and linear mixed models: age, sex, BMI, smoking, alcohol use, ADL disability, chronic conditions, and self-rated general health. All statistical analyses were conducted in Stata/*SE* 15.0 and a 2-sided *p* value less than .05 was considered statistical significance.

## Results

### Creation of Polysocial Score for Dementia

Of a total of 24 social factors included in the forward stepwise Cox regression model, 8 were retained and subsequently used for creating the polysocial score: education level, total household income, housing type, life insurance, employment status, total wealth, marital status, and neighborhood social cohesion (Table 1). The polysocial score for dementia ranged from 0 to 42; the mean (*SD*) is 23.5 (7.6). Of all included participants, 2 265 (32.6%), 2 235 (32.2%), and 2 445 (35.2%) had low (0–20), intermediate (21–27), and high (28+) polysocial score, respectively.

**Table 1.** Stepwise Cox Regression to Select Social Determinants of Health for the Polysocial Score Among Older Adults Aged 65 and Above

Social Determinants of Health	Stepwise Analysis^*^		*p* Value	Score
	Coefficient	HR (95% CI)		
Education level	—	—	—	—
Less than high school	Ref.	Ref.	—	—
High school graduate	−0.73	0.48 (0.40–0.58)	<.001	7
Postsecondary	−1.03	0.36 (0.29−0.45)	<.001	10
Total household income (U.S. dollars, $)^†^	—	—	—	—
0–20 749.28	Ref.	Ref.	—	—
20 749.28–36 032	−0.32	0.73 (0.59−0.91)	<.001	3
36 032–64 264	−0.59	0.56 (0.42−0.73)	<.001	5
64 264–471 757.2	−0.62	0.54 (0.39−0.74)	<.001	6
Housing type	—	—	—	—
Two-family house/duplex	Ref.	Ref.	—	—
Mobile house	−0.83	0.43 (0.28−0.68)	<.001	8
Apartment/Condo/townhouse/3–4 family house	−0.29	0.74 (0.56−0.98)	.04	3
One family house	−0.57	0.57 (0.45−0.72)	<.001	6
Life insurance coverage	—	—	—	—
No	Ref.	Ref.	—	—
Yes	−0.26	0.77 (0.65−0.91)	.002	3
Total wealth (U.S. dollars, $)^‡^	—	—	—	—
769 100–84 500	Ref.	Ref.	—	—
84 500–264 500	−0.33	0.72 (0.58−0.89)	.003	3
264 500–616 000	−0.35	0.71 (0.55−0.90)	.006	4
616 000–4 930 000	−0.48	0.62 (0.46−0.84)	.002	5
Employment status	—	—	—	—
Retired	Ref.	Ref.	—	—
Working	−0.45	0.64 (0.45−0.89)	.009	5
Marital status	—	—	—	—
Widowed	Ref.	Ref.	—	—
Separated/divorced	−0.40	0.67 (0.51−0.88)	.004	4
Married/Partnered	−0.08	0.92 (0.75−1.12)	.41	1
Neighborhood social cohesion^§^	—	—	—	—
1.00−4.38	Ref.	Ref.	—	—
4.38−4.75	−0.17	0.84 (0.65−1.08)	.17	2
4.75−5.75	−0.18	0.83 (0.68−1.02)	.08	2
5.75−7.00	−0.28	0.76 (0.61−0.94)	.01	3

*Notes*: ^*^Forward stepwise analysis incrementally included the education level, total household income, housing type, life insurance coverage, total wealth, employment status, marital status, discrimination, and out-of-pocket medical expenditure.

^†^Total household income was a sum of participants’ and their spouses’ earnings, pensions and annuities, Supplemental Security Income, Social Security Disability Income, Social Security retirement income, unemployment and workers’ compensation, other government transfers, household capital income, and other income (26).

^‡^Total wealth was a sum of all wealth components less debt including the net value of the first and second residence, the net value of the real estate, the net value of vehicles, the net value of businesses, the net value of individual retirement arrangement, the net value of stocks and other investments, the net value of bonds, and the net value of all other savings (26).

^§^Neighborhood social cohesion was rated by 7 conditions of the neighborhood areas within a 20-minute walk or a mile: (i) feel part of this area; (ii) no vandalism and graffiti; (iii) people can be trusted; (iv) people are afraid of walking alone in the dark; (v) most people are friendly; (vi) this area is kept very clean; (vii) people help you if in trouble; and (viii) no vacant or deserted houses. Each condition was rated by a score ranging from 1 (worst) to 7 (best) and was summed and averaged to measure overall neighborhood social cohesion.

### Sample Characteristics

At baseline, the mean age was 74.2 years (standard deviation [*SD*] = 6.8 years); 3 196 (46.0%) were male and 5 853 (84.3%) were non-Hispanic White (Table 2). Compared to participants with a low or intermediate polysocial score, those with a high polysocial score were more likely to be younger, male, and non-Hispanic White, and less likely to smoke and drink alcohol. Additionally, persons with a high polysocial score had a higher baseline cognitive score (16.7) than those in the low (13.4) and intermediate (15.2) polysocial scores categories, respectively. The most prevalent chronic diseases at baseline were arthritis (4 761 [68.6%]), hypertension (4 435 [63.9%]), heart diseases (2 139 [30.8%]), and diabetes (1 479 [21.3%]). Participants with a high polysocial score had the lowest prevalence of chronic conditions. Supplementary Table 2 also demonstrated that participants with missing data had a higher prevalence of ADL disability and poorer self-reported health, but lower hypertension prevalence compared to participants with completed data.

**Table 2.** Sample Characteristics Across Polysocial Score Categories at Baseline

Characteristics	Polysocial score categories				*p* Value ^*^
	0−20 (low)	21–27 (intermediate)	28 + (high)	Total	
	*n *= 2 265 (32.6%)	*n* = 2 235 (32.2%)	*n* = 2 445 (35.2%)	*n* = 6 945 (100.0%)	
Age, years (Mean, *SD*)	76.2 (7.5)	74.4 (6.7)	72.2 (5.6)	74.2 (6.8)	<.001
Male (*N*, %)	851 (37.6)	974 (43.6)	1 371 (56.1)	3 196 (46.0)	<.001
Race or ethnicity (*N*, %)	—	—	—	—	<.001
Non-Hispanic White	1 642 (72.5)	1 957 (87.6)	2 254 (92.2)	5 853 (84.3)	—
Non-Hispanic Black	401 (17.7%)	194 (8.7)	138 (5.6)	733 (10.6)	—
Hispanic	221 (9.8)	84 (3.8)	53 (2.2)	358 (5.2)	—
BMI category (*N*, %)^†^	—	—	—	—	.02
Normal	682 (30.3)	694 (31.3)	753 (31.1)	2 129 (30.9)	—
Overweight	864 (38.4)	905 (40.9)	1 015 (41.9)	2 784 (40.5)	—
Obese	702 (31.2)	615 (27.8)	652 (26.9)	1 969 (28.6)	—
Smoking status (*N*, %)	—	—	—	—	<.001
Never	885 (39.3)	914 (41.2)	1 030 (42.5)	2 829 (41.0)	—
Former	1 066 (47.4)	1 089 (49.1)	1 226 (50.6)	3 381 (49.0)	—
Current	299 (13.3)	217 (9.8)	169 (7.0)	685 (9.9)	—
Alcohol use (*N*, %)	—	—	—	—	<.001
0 drinks per week	1 799 (79.5)	1 522 (68.2)	1 341 (54.9)	4 662 (67.2)	—
1–2 drinks per week	242 (10.7)	308 (13.8)	449 (18.4)	999 (14.4)	—
3–4 drinks per week	77 (3.4)	137 (6.1)	217 (8.9)	431 (6.2)	—
5+ drinks per week	146 (6.4)	266 (11.9)	436 (17.8)	848 (12.2)	—
Baseline cognitive score (Mean, *SD*)	13.4 (3.6)	15.2 (3.5)	16.7 (3.3)	15.2 (3.7)	<.001
ADL difficulties (*N*, %)^‡^	556 (24.5)	282 (12.6)	187 (7.6)	1 025 (14.8)	<.001
Hypertension (*N*, %)	1 541 (68.1)	1 435 (64.3)	1 459 (59.7)	4 435 (63.9)	<.001
Diabetes (*N*, %)	617 (27.2)	460 (20.6)	402 (16.4)	1 479 (21.3)	<.001
Cancer (*N*, %)	278 (12.3)	262 (11.7)	311 (12.7)	851 (12.3)	.56
Lung disease (*N*, %)	377 (16.6)	274 (12.3)	189 (7.7)	840 (12.1)	<.001
Heart disease (*N*, %)	808 (35.7)	653 (29.2)	678 (27.7)	2 139 (30.8)	<.001
Stroke (*N*, %)	258 (11.4)	167 (7.5)	107 (4.4)	532 (7.7)	<.001
Psychiatric disease (*N*, %)	420 (18.6)	279 (12.5)	236 (9.7)	935 (13.5)	<.001
Arthritis (*N*, %)	1 650 (72.9)	1 567 (70.1)	1 544 (63.1)	4 761 (68.6)	<.001
Self-reported health (*N*, %)	—	—	—	—	<.001
Excellent/very good	581 (25.7)	916 (41.0)	1 320 (54.0)	2 817 (40.6)	—
Good	729 (32.2)	794 (35.6)	782 (32.0)	2 305 (33.2)	—
Fair/poor	953 (42.1)	522 (23.4)	341 (14.0)	1 816 (26.2)	—

*Notes*: ADL = activities of daily living; BMI = body mass index.

^*^Obtained from the analysis of variance tests for continuous covariates or chi-square tests for categorical covariates.

^†^Underweight or normal (BMI ≤24.9 kg/m^2^), overweight (BMI 25.0–30.0 kg/m^2^), and obese (BMI ≥ 30.0 kg/m^2^).

^‡^Difficulties in performing one of the 5 ADLs (bathing, dressing, eating, getting in and out of bed, and using the toilet).

### Polysocial Score and Incident Dementia

**Figure 1.**
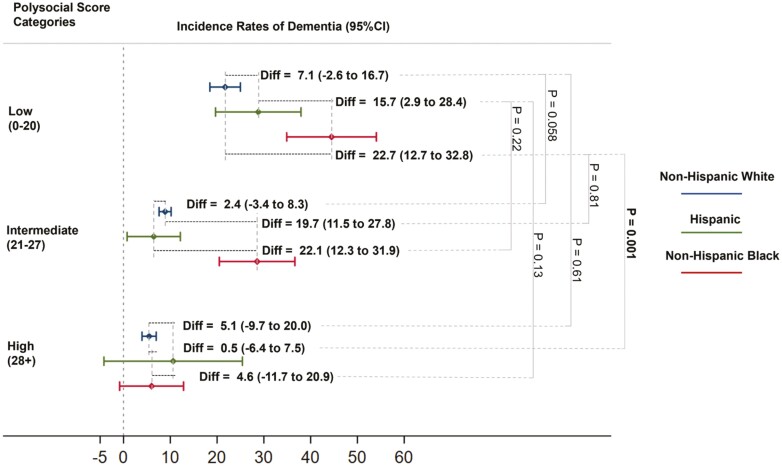
The incidence rate of dementia by polysocial categories and race or ethnicity among community-dwelling older adults aged 65 years or above. CI = confidential interval. The *p* value was calculated by the interaction method in the Poisson regression models. These results were adjusted for age, sex, lifestyles (body mass index, smoking status, and alcohol use), and health measures (disability, hypertension, diabetes, cancer, lung disease, heart disease, stroke, psychiatric disease, arthritis, and self-reported health).

The incidence rates of dementia were 25.2, 10.1, and 4.6 per 1 000 PYs among participants in low, intermediate, and high polysocial score categories, respectively (Table 3). Similar trends of incidence rates of dementia were observed for non-Hispanic White (21.2, 8.8, and 4.4 per 1 000 PYs), non-Hispanic Black (40.2, 23.9, and 8.1 per 1 000 PYs), and Hispanic (27.2, 7.3, and 4.2 per 1 000 PYs) older people, separately. The dementia rate among non-Hispanic Black participants with low polysocial score was 18.9 (95% CI: 10.8 to 27.1) and 13.0 (95% CI: 1.9 to 24.2) per 1 000 PYs higher than their non-Hispanic White and Hispanic counterparts, respectively. These differences substantially attenuated and were no longer significant among persons with a high polysocial score (3.6 [95% CI: −1.7 to 9.0] and 3.9 [95% CI: −4.0 to 11.7] per 1 000 PYs, respectively). The difference in dementia incidence rate between non-Hispanic Black and non-Hispanic White older people was significantly lower in the high polysocial score category than those in the low one (15.3 per 1 000 PYs; *p* = .006). We observed similar results for the comparison between non-Hispanic Black and Hispanic persons, though the difference was not significant (9.1 per 1 000 PYs; *p* = .42). The difference in dementia rate was small between non-Hispanic White and Hispanic participants across all polysocial score categories. These patterns persisted after multivariable adjustment (Figure 1).

**Table 3.** The Incidence Rates of Dementia by Polysocial Score Categories and Race or Ethnicity Among Community-Dwelling Older Adults Aged 65 Years or Above

Variable	Polysocial Score Categories			Score Comparison		
	Low (0–20)	Intermediate (2–27)	High (28 +)	Low Versus High	Low Versus Intermediate	Intermediate Versus High
	Dementia Cases Per 1 000 PYs (95% CI)			Dementia Cases Per 1 000 PYs (*p* Value)		
Overall	25.2 (22.8 to 27.9)	10.1 (8.7 to 11.7)	4.6 (3.8 to 5.7)			
Race or ethnicity	—	—	—			
Non-Hispanic White	21.2 (18.7 to 24.2)	8.8 (7.4 to 10.5)	4.4 (3.6 to 5.5)			
Non-Hispanic Black	40.2 (33.2 to 48.6)	23.9 (17.2 to 33.4)	8.1 (4.2 to 15.5)			
Hispanic	27.2 (20.1 to 36.6)	7.3 (3.1 to 17.6)	4.2 (1.0 to 16.7)			
Risk difference in race or ethnicity	—	—	—			
Non-Hispanic Black vs non-Hispanic White	18.9 (10.8 to 27.1)	15.1 (7.0 to 23.2)	3.6 (−1.7 to 9.0)			
Hispanic vs non-Hispanic White	5.9 (−2.7 to 14.5)	1.5 (−5.1 to 8.1)	0.3 (−5.6 to 6.1)			
Non-Hispanic Black vs Hispanic	13.0 (1.9 to 24.2)	16.6 (6.4 to 26.8)	3.9 (−4.0 to 11.7)			
Comparison of risk differences in race or ethnicity						
Comparison of risk difference^*^ (non-Hispanic Black vs non-Hispanic White)				15.3 (0.006)	3.5 (0.75)	11.5 (0.03)
Comparison of risk difference^*^ (Hispanic vs non-Hispanic White)				5.6 (0.18)	4.3 (0.12)	1.2 (0.81)
Comparison of risk difference^*^ (non-Hispanic Black vs Hispanic)				9.1 (0.42)	−3.6 (0.37)	12.7 (0.006)

*Notes*: CI = confidential interval; PYs = person-years.

^*^Comparison of the risk difference in race or ethnicity between 2 polysocial score categories was calculated by the interaction between polysocial score categories and race or ethnicity groups in the unadjusted Poisson model.

### Polysocial Score and Cognitive Decline

**Figure 2.**
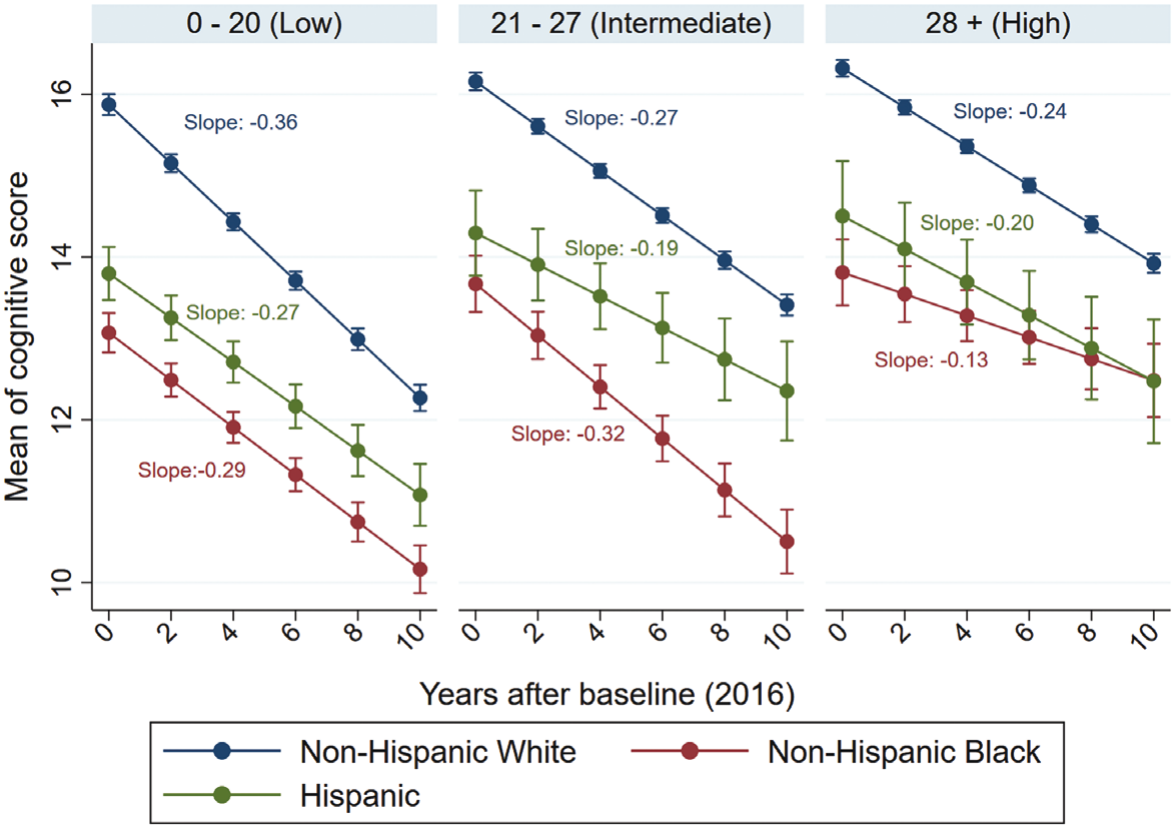
Marginal means and 95% confidence interval of the cognitive function by polysocial score categories among community-dwelling older adults aged 65 years or above. The linear mixed model was adjusted for age, sex, lifestyles (body mass index, smoking status, and alcohol use), and health measures (ADL disability, hypertension, diabetes, cancer, lung disease, heart disease, stroke, psychiatric disease, arthritis, and self-reported health).

Figure 2 showed the estimated decline in cognitive function by polysocial score categories and race or ethnicity. After multivariable adjustment, the 10-year average decline in cognitive score was 3.6 among non-Hispanic White older adults in the low polysocial score category, which was 0.9 (33.3%) higher than Hispanic (difference: −0.9 [33.3%]; see Supplementary Table 3) and 0.7 (24.1%) higher than non-Hispanic Black older persons (difference: −0.7 [24.1%]). Among persons in the intermediate polysocial category, the estimated 10-year decline in cognitive score was 2.7 among non-Hispanic White older adults and 3.2 among non-Hispanic Black seniors. The predicted cognitive decline then attenuated to 2.4, 1.3, and 2.0 among non-Hispanic White, non-Hispanic Black, and Hispanic older participants in the high polysocial score category.

## Discussion and Implications

A polysocial score for incident dementia was built by using a nationally representative sample of U.S. older adults aged 65 or above, comprising 8 social determinants of health reflecting individual and community level of social and physical environments. Older adults with a higher polysocial score had a lower dementia incidence and slower cognitive decline than those with an intermediate and low polysocial score. Non-Hispanic Black older adults in the low polysocial score category had a substantially higher incidence of dementia than non-Hispanic White and Hispanic older adults, whereas these differences largely attenuated among participants in the high polysocial score category, particularly between non-Hispanic Black and non-Hispanic White older adults.

Our results were consistent with previous studies evaluating the associations between social factors and the incidence of dementia among community-dwelling older adults (27,28). A longitudinal study in the UK demonstrated that older adults with less wealth, more neighborhood deprivation, and lower education were associated with a higher incidence of dementia (27). A systematic review documented consistent evidence that a lower education level was associated with a greater risk of dementia in later life in developed regions (28). Our polysocial score approach identified that baseline education level was the strongest component affecting the incidence of dementia among older adults aged 65 and above in the United States. This finding was also reflected by another longitudinal study in the United States, showing that older adults with high school education levels were associated with the largest positive effect on memory (29).

We found that non-Hispanic Black older persons had a substantially higher incidence of dementia than non-Hispanic White and Hispanic older adults, which was in line with prior studies that have consistently shown a higher burden of dementia among racial and ethnic minorities, especially among non-Hispanic Black older adults (4–6,30). Several social determinants of health, such as education, wealth, and household income, were shown to explain the racial and ethnic disparities in dementia (8,9). More years of education and better early-life education quality were associated with attenuated disparity of cognitive decline between non-Hispanic Black and non-Hispanic White older adults (9,10). Wealth and household income could also attenuate the disproportionate risk of dementia between non-Hispanic White and non-Hispanic Black older people (8). Our results revealed that the racial and ethnic difference in dementia incidence between non-Hispanic White and non-Hispanic Black older adults was largely attenuated among persons with high polysocial score. Given that the stronger contributors of this polysocial score are related to individual and household wealth such as education and household income, possible explanation of this moderation effect of better polysocial score on dementia risk between non-Hispanic Black and White older adults is that older persons with better social status may have better physical and mental health and have access to proper medical care, which may moderate this racial or ethnic disparity of dementia risk (31). Additionally, this attenuated disparity of dementia risk may also be explained by the better social connections and social support among older adults. Studies demonstrated that older persons with better socioeconomic status had more social support and larger social network (32,33), which may reduce the risk of cognitive impairment (34).

This finding highlighted the significance of the presented polysocial score approach in understanding how multiple social factors, which are multidimensional and often interconnected, collectively contribute to health disparities among older adults with distinct races or ethnicities in the United States.

We found that the incidence rate of dementia among Hispanic older adults did not significantly differ from that among non-Hispanic White older participants in all 3 polysocial score categories after adjusting for demographic, lifestyle, and health conditions. The potential explanation may be related to the “Hispanic Paradox” that Hispanic Americans had a healthier physical condition compared to non-Hispanic White persons in the United States (35). Despite that this immigrant health effect was mainly restricted to mortality, one cross-sectional study delineated that foreign-born Mexican Americans were associated with a similar probability of cognitive impairment to non-Hispanic White middle-aged older adults in the United States (36). Another cross-sectional study evaluated the moderation effect of education and health literacy on the racial or ethnic difference in cognitive impairment, revealing that foreign-born Hispanic middle-aged adults had similar risk of cognitive impairment to non-Hispanic White middle-aged adults with equal education levels (9). This evidence may imply that Hispanic older adults who remain living in the United States had a similar or better health status than most American older people, regardless of their difference in social status.

This study has several strengths. First, this study utilized a large and nationally representative sample of community-dwelling older adults aged 65 and above with comprehensive inclusion of a wide array of social determinants of health in the United States. Second, this prospective cohort study with a 10-year follow-up period allowed us to measure the long-term effect of multiple social determinants of health on both dementia incidence and the temporal cognitive decline of older adults in the United States. Third, the construction of the polysocial score that included 5 dimensions of social determinants of health may broaden the consideration of individual socioeconomic factors by covering the neighborhood environment around older adults. Related, this composite score may further enrich the evidence in seeking strong social determinants of health to potentially reduce the racial or ethnic disparity of dementia between non-Hispanic Black and non-Hispanic White older populations in the United States. This study is not without limitations. First, the present study had a relatively small sample size of non-Hispanic Black and Hispanic cohorts, which restricted our statistical power to conduct further stratified analysis. Second, we only included data on social determinants of health at baseline so the longitudinal variation of social determinants of health in our cohort was overlooked. Future research is needed to characterize the temporal changes in polysocial score and examine how the variations contribute to health among older adults. Third, the development of polysocial score for dementia among older adults may need external validation from a distinct data set in the United States, though our previous work on developing polysocial score for mortality showed the internal validity of this approach (18).

In summary, we created a polysocial score to capture the aggregated effects of 8 social determinants of health on the risk of dementia incidence among older adults aged 65 and above in the United States. Older people with a higher polysocial score had a lower incidence rate of dementia and a slower rate of cognitive. The racial and ethnic difference found in the low polysocial score category was largely attenuated among persons with a high polysocial score, particularly between non-Hispanic Black and non-Hispanic White older participants. The novel polysocial score approach provided an excellent opportunity to comprehensively understand and address racial and ethnic disparities in dementia risk among older Americans. This novel tool, quantifying the effect of social determinants of health on dementia, may be integrated into existing dementia prevention programs to identify community-dwelling older adults with distinct aspects of social disadvantages and provide them with tailored interventions for dementia prevention.

## Supplementary Material

igae078_suppl_Supplementary_Materials

## Data Availability

This article did not contain any individual data in any form. Availability of data and materials: Data from the 2006 to 2016 waves of the Health and Retirement Study were open-accessed from the following link: https://hrs.isr.umich.edu/documentation/data-descriptions.
