# Supplementary material for: A Polysocial Approach in Exploring Racial and Ethnic Differences in Dementia and Cognitive Decline Among U.S. Older Adults: Health and Retirement Study
Source: Innov Aging. 2024 Aug 28;8(10):igae078. doi: 10.1093/geroni/igae078 (PMC11481015; doi:10.1093/geroni/igae078)
Supplement: igae078_suppl_Supplementary_Materials [file igae078_suppl_supplementary_materials.docx]

***Innovation in Aging* Supplementary Material: Ping et al. A Polysocial Approach in Exploring Racial and Ethnic Differences in Dementia and Cognitive Decline among Community-dwelling Older Adults in the US: Findings from the Health and Retirement Study**.

**Supplementary Figure 1.** Flow diagram of the participant selection process

**
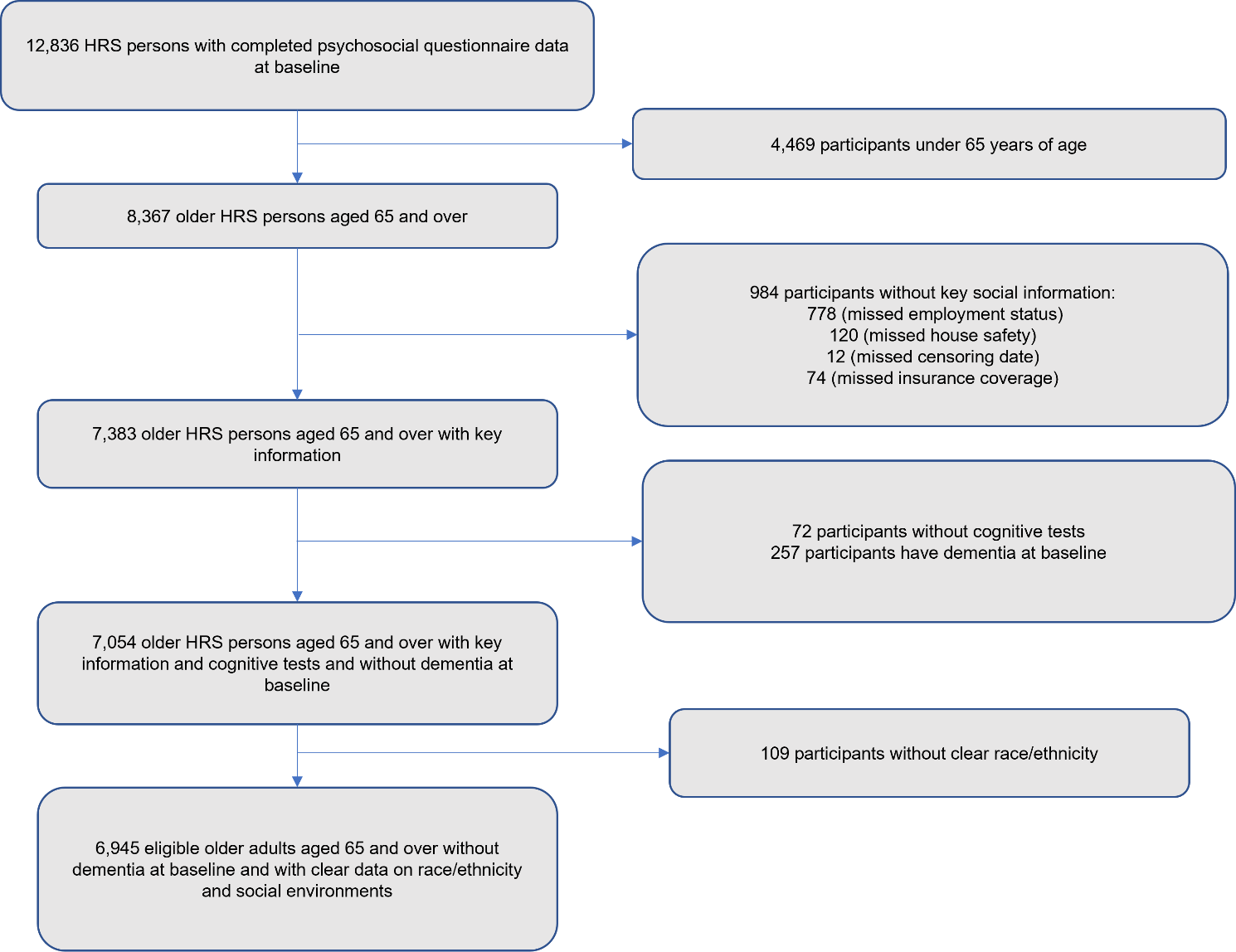
**

**Abbreviation:** HRS, the Health and Retirement Study.

**Supplementary Table 1.** Categories of all 24 social determinants of health

| Social determinants of health | Observations (N, %) | Interpretations of Social determinants of health |
| --- | --- | --- |
| Annual individual income ^a^, $ |  |  |
| 0 - 10560 | 1738 (25.0) | Annual individual income: 0 – 10560 thousand dollars |
| 10560 - 16800 | 1747 (25.2) | Annual individual income: 10560 – 16800 thousand dollars |
| 16800 - 28800 | 1734 (25.0) | Annual individual income: 16800– 28800 thousand dollars |
| 28800 - 160000 | 1726 (24.9) | Annual individual income: 28800 – 160000 thousand dollars |
| Total household income ^b^, $ |  |  |
| 0 - 20749.28 | 1737 (25.0) | Total household income: 0 – 20749.28 thousand dollars |
| 20749.28 - 36032 | 1736 (25.0) | Total household income: 20749.28 – 36032 thousand dollars |
| 36032 - 64264 | 1736 (25.0) | Total household income: 36032 – 64264 thousand dollars |
| 64264 - 471757.2 | 1736 (25.0) | Total household income: 64264 – 471757.2 thousand dollars |
| Total wealth ^c^ |  |  |
| -769100 - 84500 | 1739 (25.0) | The net value of total wealth: -769100 – 84500 thousand dollars |
| 84500 - 264500 | 1734 (25.0) | The net value of total wealth: 84500 – 264500 thousand dollars |
| 264500 - 616000 | 1736 (25.0) | The net value of total wealth: 264500 – 616000 thousand dollars |
| 616000 - 4930000 | 1736 (25.0) | The net value of total wealth: 616000 – 4930000 thousand dollars |
| Total non-housing wealth ^d^, $ |  |  |
| -196500 - 15000 | 1768 (25.5) | The net value of non-housing wealth: -196500 – 15000 thousand dollars |
| 15000 - 96700 | 1706 (24.6) | The net value of non-housing wealth: 15000 – 96700 thousand dollars |
| 96700 - 365000 | 1741 (25.1) | The net value of non-housing wealth: 96700 – 365000 thousand dollars |
| 365000 - 4121460 | 1730 (24.9) | The net value of non-housing wealth: 365000 – 4121460 thousand dollars |
| Total debts ^e^ |  |  |
| No debts | 3946 (56.8) | Respondents had no debts |
| With debts | 2999 (43.2) | Respondents had debts: 0 – 450000 thousand dollars |
| Out-of-pocket medical expenditure ^f^, $ |  |  |
| 0 - 596 | 1739 (25.0) | Annual out-of-pocket medical expenditure: 0 – 596 thousand dollars |
| 596 - 1560 | 1737 (25.0) | Annual out-of-pocket medical expenditure: 596 – 1560 thousand dollars |
| 1560 - 3450 | 1738 (25.0) | Annual out-of-pocket medical expenditure:1560 – 3450 thousand dollars |
| 3450 - 38300 | 1731 (24.9) | Annual out-of-pocket medical expenditure: 3450 – 38300 thousand dollars |
| Total housing value ^g^, $ | ·· | ·· |
| -795000 - 40900 | 1737 (25.0) | The net value of total housing value: -795000 – 40900 thousand dollars |
| 40900 - 125000 | 1789 (25.8) | The net value of total housing value: 40900 – 125000 thousand dollars |
| 125000 - 250000 | 1760 (25.3) | The net value of total housing value: 125000 – 250000 thousand dollars |
| 250000 - 1250000 | 1659 (23.9) | The net value of total housing value: 250000 – 1250000 thousand dollars |
| Marital Status | ·· | ·· |
| Widowed | 1676 (24.1) | Respondents were widowed |
| Separated/Divorced | 790 (11.4) | Respondents were separated, divorced, or never married |
| Married/Partnered | 4479 (64.5) | Respondents were married, married but spouse absent, or partnered |
| Education level |  |  |
| Less than high school | 1589 (22.9) | Respondents completed 1-11 years of education |
| High school graduate | 2397 (34.5) | Respondents completed 12 years of education |
| Postsecondary | 2959 (42.6) | Respondents completed more than 12 years of education |
| Employment status | ·· | ·· |
| Retired | 6173 (88.9) | Labor status: partly retired or retired |
| Working | 772 (11.1) | Labor status: work full-time or part-time |
| Housing types | ·· | ·· |
| Two-family house/duplex | 680 (9.9) | Respondents lived in a two-family house/duplex |
| Mobile house | 318 (4.6) | Respondents lived in a mobile house |
| Apartment/Condo/townhouse/3-4 family house | 1147 (16.7) | Respondents lived in a three-four family house, apartment, condominium, or townhouse. |
| One-family house | 4734 (68.8) | Respondents lived in a one-family house |
| Type of residence | ·· | ·· |
| Rural | 2262 (32.6) | Respondents lived in the rural area |
| Urban | 4669 (67.4) | Respondents lived in the urban area |
| Region of residence | ·· | ·· |
| South | 2756 (39.7) | Respondents lived in the south region of America |
| Northeast | 1090 (15.7) | Respondents lived in the northeast region of America |
| Midwest | 1844 (26.6) | Respondents lived in the Midwest region of America |
| West | 1248 (18.0) | Respondents lived in the west region of America |
| Neighborhood safety rate | ·· | ·· |
| Fair | 547 (7.9) | Self-reported neighborhood safety condition: poor or fair |
| Good | 1388 (20.0) | Self-reported neighborhood safety condition: good |
| Very good | 2334 (33.6) | Self-reported neighborhood safety condition: very good |
| Excellent | 2676 (38.5) | Self-reported neighborhood safety condition: excellent |
| Interviewed language | ·· | ·· |
| English | 6793 (97.8) | Native language: English |
| Spanish | 152 (2.2) | Native language: Spanish |
| Healthcare insurance coverage | ·· | ·· |
| Medicare/Medicaid only | 2860 (41.5) | Respondents were covered by government insurance only |
| Medicare/Medicaid and private insured | 4038 (58.5) | Respondents were covered both by government insurance and private insurance |
| Long-term care insurance coverage | ·· | ·· |
| No | 5846 (84.2) | Respondents were not covered by long-term care insurance |
| Yes | 1099 (15.8) | Respondents were covered by long-term care insurance |
| Life insurance coverage | ·· | ·· |
| No | 2361 (34.2) | Respondents were not covered by life insurance |
| Yes | 4537 (65.8) | Respondents were covered by life insurance |
| Care satisfaction | ·· | ·· |
| Not satisfied | 2648 (38.2) | Self-rated healthcare quality: no satisfied or somewhat satisfied |
| Satisfied | 4277 (61.8) | Self-rated healthcare quality: very satisfied |
| Social engagement | ·· | ·· |
| Low | 2737 (39.4) | Social engagement score: 0 - 1 |
| Moderate | 3442 (49.6) | Social engagement score: 2 - 3 |
| High | 766 (11.0) | Social engagement score: 4 - 5 |
| Discrimination ^h^ | ·· | ·· |
| 0.00 - 4.20 | 2199 (31.7) | Daily discrimination: 0 - 4.2 |
| 4.20 - 4.60 | 1410 (20.3) | Daily discrimination: 4.2 - 4.6 |
| 4.60 - 5.00 | 3336 (48.0) | Daily discrimination: 4.6 - 5 |
| Stress level | ·· | ·· |
| 3 and more events | 2013 (29.0) | Stressful events happened 3 or more times previously |
| 2 events | 1588 (22.9) | Stressful events happened 2 times previously |
| 1 event | 1890 (27.2) | Stressful events happened 1 time previously |
| 0 event | 1454 (20.9) | Stressful events never happened previously |
| Neighborhood social cohesion | ·· | ·· |
| 1.00 - 4.38 | 1678 (24.2) | Neighborhood social cohesion score: 1 - 4.375 |
| 4.38 - 4.75 | 1157 (16.7) | Neighborhood social cohesion score: 4.375 - 4.75 |
| 4.75 - 5.75 | 2217 (31.9) | Neighborhood social cohesion score: 4.75 - 5.75 |
| 5.75 - 7.00 | 1893 (27.3) | Neighborhood social cohesion score: 5.75 - 7 |
| Social support | ·· | ·· |
| 0.00 - 1.75 | 1864 (26.8) | Social support score: 0 - 1.75 |
| 1.75 - 2.10 | 1824 (26.3) | Social support score: 1.75 - 2.10 |
| 2.10 - 2.45 | 1464 (21.1) | Social support score: 2.10 - 2.45 |
| 2.45 - 3.00 | 1793 (25.8) | Social support score: 2.45 – 3 |

^a^ Annual individual income was a sum of participants' earnings, income from SDI and SSI, unemployment compensations, and other government transfers.^36^

^b^ Total household income was a sum of participants’ and their spouses’ earnings, pensions and annuities, Supplemental Security Income (SSI), Social Security Disability Income (SDI), Social Security retirement income, unemployment and workers’ compensation, other government transfers, household capital income, and other income.^36^

^c^ Total wealth was a sum of all wealth components less debt including the net value of the first and second residence, the net value of the real estate, the net value of vehicles, the net value of businesses, the net value of individual retirement arrangement (IRA), the net value of stocks and other investments, the net value of bonds, and the net value of all other savings.^36^

^d^ Total non-housing wealth was a sum of the net value of the real estate, the net value of vehicles, net value of businesses, net value of IRA, the net value of stocks and other investments, the net value of bonds, and the net value of all other savings.^36^

^e^ Total debt was a sum of the first and second house mortgages, home equity line of credit balances, home equity loans, credit card balances, medical debts, life insurance policy loans, and loans from relatives.^36^

^f^ Out-of-pocket medical expenditure was a sum of total out-of-pocket medical expenditure from participants and spouses or partners.^36^

^g^ Total housing value was a sum of the net value of the primary residence and the net value of the secondary residence.^36^

^h^ Discrimination was reverse coded, and a higher score indicated less discrimination.

**Supplementary Table 2.** The comparison between participants with completed and missing data.

| **Characteristics** | **With Completed data** | **With missing data** | **Total** | **P-value** |
| --- | --- | --- | --- | --- |
|  | N=6556 | N=389 | N=6945 |  |
| Age, years (Mean, SD) | 74.2 (6.8) | 73.9 (7.7) | 74.2 (6.8) | 0.41 |
| Male (N, %) | 3020 (46.1) | 176 (45.2) | 3196 (46.0) | 0.75 |
| Race and Ethnicity (N, %) | .. | .. | .. | 0.10 |
| Non-Hispanic White | 5534 (84.4) | 319 (82.2) | 5853 (84.3) | .. |
| Non-Hispanic Black | 693 (10.6) | 40 (10.3) | 733 (10.6) | .. |
| Hispanic | 329 (5.0) | 29 (7.5) | 358 (5.2) | .. |
| BMI Category (N, %) ^a^ | .. | .. | .. | 0.41 |
| Normal | 2039 (31.1) | 90 (27.6) | 2129 (30.9) | .. |
| Overweight | 2646 (40.4) | 138 (42.3) | 2784 (40.5) | .. |
| Obese | 1871 (28.5) | 98 (30.1) | 1969 (28.6) | .. |
| Smoking status (N, %) | .. | .. | .. | 0.29 |
| Never | 2704 (41.2) | 125 (36.9) | 2829 (41.0) | .. |
| Former | 3204 (48.9) | 177 (52.2) | 3381 (49.0) | .. |
| Current | 648 (9.9) | 37 (10.9) | 685 (9.9) | .. |
| Alcohol use (N, %) | .. | .. | .. | 0.28 |
| 0 drinks per week | 4399 (67.1) | 263 (68.5) | 4662 (67.2) | .. |
| 1-2 drinks per week | 949 (14.5) | 50 (13.0) | 999 (14.4) | .. |
| 3-4 drinks per week | 414 (6.3) | 17 (4.4) | 431 (6.2) | .. |
| 5+ drinks per week | 794 (12.1) | 54 (14.1) | 848 (12.2) | .. |
| Baseline cognitive score (Mean, SD) | 15.2 (3.7) | 15.1 (3.8) | 15.2 (3.7) | 0.72 |
| ADL difficulties (N, %) ^b^ | 945 (14.4) | 80 (20.6) | 1025 (14.8) | 0.0010 |
| Hypertension (N, %) | 4214 (64.3) | 221 (57.9) | 4435 (63.9) | 0.011 |
| Diabetes (N, %) | 1401 (21.4) | 78 (20.1) | 1479 (21.3) | 0.55 |
| Cancer (N, %) | 804 (12.3) | 47 (12.1) | 851 (12.3) | 0.93 |
| Lung disease (N, %) | 795 (12.1) | 45 (11.7) | 840 (12.1) | 0.80 |
| Heart disease (N, %) | 2040 (31.1) | 99 (25.6) | 2139 (30.8) | 0.022 |
| Stroke (N, %) | 495 (7.6) | 37 (9.6) | 532 (7.7) | 0.15 |
| Psychiatric disease (N, %) | 873 (13.3) | 62 (16.1) | 935 (13.5) | 0.13 |
| Arthritis (N, %) | 4508 (68.8) | 253 (65.4) | 4761 (68.6) | 0.16 |
| Self-reported Health (N, %) | .. | .. | .. | 0.011 |
| Excellent/Very Good | 2675 (40.8) | 142 (37.2) | 2817 (40.6) | .. |
| Good | 2190 (33.4) | 115 (30.1) | 2305 (33.2) | .. |
| Fair/Poor | 1691 (25.8) | 125 (32.7) | 1816 (26.2) | .. |

**Abbreviations**: ADL, activities of daily living; BMI, body mass index.

^a^ Underweight or normal (BMI$\leq$24.9 kg/m^2^), overweight (BMI 25.0-30.0 kg/m^2^), and obese (BMI greater$\geq$30.0 kg/m^2^).

^b^ Difficulties in performing one of the five ADLs (bathing, dressing, eating, getting in and out of bed, and using the toilet).

**Supplementary Table 3**. Race-specific longitudinal association between polysocial score and cognitive decline.

| **Race and Ethnicity** | **Cognitive Decline (95%CI) ^a^** | | |
| --- | --- | --- | --- |
|  | **Polysocial Score** | | |
|  | **Low**  **(0 - 20)** | **Intermediate**  **(21 - 27)** | **High**  **(28 +)** |
| Non-Hispanic White | -3.6 (-3.8 to -3.4) | -2.7 (-2.9 to -2.6) | -2.4 (-2.5 to -2.3) |
| Non-Hispanic Black | -2.9 (-3.3 to -2.5) | -3.2 (-3.7 to -2.6) | -1.3 (-1.9 to -0.8) |
| Hispanic | -2.7 (-3.2 to -2.2) | -1.9 (-2.7 to -1.2) | -2.0 (-3.0 to -1.0) |
| Difference, n (%) ^b^  (Non-Hispanic White vs. non-Hispanic Black) | -0.7 (24.1) | 0.5 (-15.6) | -1.1 (84.6) |
| Difference, n (%) ^b^  (Non-Hispanic White vs. Hispanic) | -0.9 (33.3) | -0.8 (42.1) | -0.4 (20.0) |
| Difference, n (%) ^b^  (Non-Hispanic Black vs. Hispanic) | -0.2 (7.4) | -1.3 (68.4) | 0.7 (-35.0) |

**Abbreviations**: CI, Confidence Interval

**Note**: These results were generated from the linear mixed model, adjusting for age, sex, lifestyles (body mass index, smoking status, and alcohol use), and health measures (ADL disability, hypertension, diabetes, cancer, lung disease, heart disease, stroke, psychiatric disease, arthritis, and self-reported health).

^a^ Cognitive decline was calculated as [the estimated mean of the cognitive score at baseline - the estimated mean of cognitive score in year 10].

^b^ The racial/ethnic difference was calculated as [the cognitive decline of A racial/ethnic group - the cognitive decline of B racial/ethnic group]. The percentage of racial and ethnic difference was calculated as [the cognitive decline of A racial/ethnic group - the cognitive decline of B racial/ethnic group] / [the cognitive decline of B racial/ethnic group]. For example, the 24.1% difference between non-Hispanic White and non-Hispanic Black older participants means that the cognitive decline in non-Hispanic White is 24.1% greater than that in the non-Hispanic Black older adults.
